# Likelihood based observability analysis and confidence intervals for predictions of dynamic models

**DOI:** 10.1186/1752-0509-6-120

**Published:** 2012-09-05

**Authors:** Clemens Kreutz, Andreas Raue, Jens Timmer

**Affiliations:** 1 Physics Department, University of Freiburg, Hermann Herder Straße 3, 79104 Freiburg, Germany; 2 Freiburg Centre for Biosystems Analysis (ZBSA), University of Freiburg, Habsburgerstraße 49, 79104 Freiburg, Germany; 3 Freiburg Institute for Advanced Studies (FRIAS), University of Freiburg, Albertstraße 19, 79104 Freiburg, Germany; 4 Freiburg Initiative in Systems Biology (FRISYS), University of Freiburg, Schaenzlestraße 1, 79104 Freiburg, Germany; 5 BIOSS Centre for Biological Signalling Studies, University of Freiburg, Schaenzlestraße 18, 79104 Freiburg; 6 Department of Clinical and Experimental Medicine, Universitetssjukhuset, 58183 Linköping, Sweden; 7 Institute of Bioinformatics and Systems Biology, Helmholtz Zentrum München, Ingolstädter Landstraße 1, 85764 Neuherberg, Germany

**Keywords:** Confidence intervals, Identifiability, Likelihood, Parameter estimation, Prediction, Profile likelihood, Optimal experimental design, Ordinary differential equations, Signal transduction, Statistical inference, Uncertainty

## Abstract

**Background:**

Predicting a system’s behavior based on a mathematical model is a primary task in Systems Biology. If the model parameters are estimated from experimental data, the parameter uncertainty has to be translated into confidence intervals for model predictions. For dynamic models of biochemical networks, the nonlinearity in combination with the large number of parameters hampers the calculation of prediction confidence intervals and renders classical approaches as hardly feasible.

**Results:**

In this article reliable confidence intervals are calculated based on the *prediction profile likelihood*. Such prediction confidence intervals of the dynamic states can be utilized for a data-based observability analysis. The method is also applicable if there are non-identifiable parameters yielding to some insufficiently specified model predictions that can be interpreted as *non-observability*. Moreover, a *validation profile likelihood* is introduced that should be applied when noisy validation experiments are to be interpreted.

**Conclusions:**

The presented methodology allows the propagation of uncertainty from experimental to model predictions. Although presented in the context of ordinary differential equations, the concept is general and also applicable to other types of models. Matlab code which can be used as a template to implement the method is provided at http://www.fdmold.uni-freiburg.de/∼ckreutz/PPL.

## Background

A major goal of Systems Biology is the prediction of cellular behavior over a broad range of environmental conditions. To be able to generate realistic predictions, the individual processes of a system of interest have to be translated into a mathematical framework. The task of establishing a realistic mathematical model which is able to reliably predict a systems behavior is to comprehensively use the existing knowledge, e.g. in terms of experimental data, to adjust the models’ structures and parameters.

The major steps of this mathematical modeling process comprise model discrimination, i.e. identification of an appropriate model structure, model calibration, i.e. estimation of unknown model parameters, as well as prediction and model validation. For all these topics it is essential to have appropriate methods assessing the certainty or ambiguity of any result for given experimental information.

For parameter estimation, there are several approaches to derive confidence intervals, like standard errors which are based on an estimate of the covariance matrix of the parameter estimates [1], bootstrap based confidence intervals [2-4], as well as likelihood based confidence intervals [5,6]. For model discrimination, significance statements can be obtained by statistical tests. However, for model predictions, there are still demands for methodology that is applicable for mathematical models like ordinary differential equations (ODEs) used to describe the dynamics of a system in a variety of scientific fields e.g. in molecular biology [7,8], but also in medical research, chemistry, engineering, and physics.

The mere estimation of parameters is often not the final aim of an investigation. More frequently, it is desired to utilize the parametrized model to generate model predictions such as the dynamic behavior of unobserved components. Classically, the uncertainty in the model parameters is attempted to be translated into corresponding prediction confidence intervals, also called *predictive intervals* or *prediction intervals* in the literature. For models that depend linearly on the model parameters, as it occurs in classical regression models, this is well studied and known as propagation of uncertainty based on standard errors. This approach is appropriate and sufficient for many applications. However, e.g. for biochemical networks, the model responses depend nonlinearly on the model parameters. Here, the boundaries of the parameter confidence region can exhibit arbitrarily complex shape and are usually difficult to translate into boundaries for the prediction confidence intervals. Therefore, established approaches aim to scan the entire parameter subspace which is in a sufficient agreement with the experimental data to propagate the parameters confidence regions into confidence intervals for the model predictions. The major challenge is the complex nonlinear interrelation between parameters and model responses which requires that the parameter space has to be sampled densely to capture all scenarios of model predictions. For models with tens to hundreds of parameters this is numerically demanding or even infeasible because high dimensional spaces cannot be sampled densely. This issue often referred to the *curse of dimensionality* in literature [9,10].

Methods for an approximate sampling of the parameter space, e.g. the Markov Chain Monte Carlo (MCMC) methods [11,12], and bootstrap based approaches [4,13] are numerically demanding and provide only rough approximations for ODE models. Therefore, it is difficult to control the coverage of the prediction confidence intervals for such approaches. Moreover non-identifiable parameters are not explicitly considered hampering the convergence of such sampling techniques and yielding results that are questionable and difficult to interpret [14].

The idea of the *prediction profile likelihood* presented here is to determine prediction confidence without an explicit sampling strategy for the parameter space. Instead, a certain fixed value for a prediction is used as a nonlinear constraint and the parameter values are chosen via constraint optimization of the likelihood. This does neither require a unique solution in terms of parameter identifiability nor confidence intervals for the parameter estimates. The constraint maximum likelihood approach checks the agreement of a predicted value with the experimental data. By repeating this procedure for continuous variations of the predicted value, the *prediction profile-likelihood* is obtained. Thresholding the prediction profile likelihood yields statistically accurate confidence intervals. The desired level of confidence which coincides with the level of agreement with the experiments is controlled by the threshold.

The theoretical background of the prediction profile likelihood, also called *predictive likelihood* has been already studied [15]. Moreover, related ideas are already applied in the context of generalized linear mixed models [16], unobserved data points [17]. The linear approximation has been applied in nonlinear regression analyses [18]. A review of prediction profile likelihood approaches and a modification to sufficiency-based predictive likelihood is provided in [19].

In this paper, this concept is applied to ODE models occurring in dynamic models, e.g. in Molecular and Systems Biology as well as chemical engineering. In this context the approach a data-based observability analysis is introduced. Moreover, the prediction profile likelihood concept is extended to obtain confidence intervals for validation experiments.

## Methods

The methodology presented in the following is general, i.e. not only applicable for ODEs. Therefore, we first introduce the prediction profile likelihood as well as prediction confidence intervals and next illustrating the applicability for ODE models.

### The prediction profile likelihood

For additive Gaussian noise *ε *∼* N*(0,*σ*^2^) with known variance *σ*^2^, two times the negative log-likelihood

(1) $$-2\;\text{LL}(y|\theta )=\underset{i}{\sum }\frac{{\left(y_{i}-F(t_{i},u,\theta )\right)}^{2}}{\sigma^{2}}+\text{const.}$$

of the data *y* for the parameters *θ *is, except a constant offset, identical to the residual sum of squares RSS(*θ*|*y*) = ∑_*i*_(*y*_*i *_−* F*(*t*_*i*_,*u*,*θ*))^2^/*σ*^2^. In this case, maximum likelihood estimation is equivalent to standard least-squares estimation

(2) $$\hat{\theta }=\text{arg}\;\underset{\theta }{\text{max}}\;\text{LL}(y|\theta )\equiv \text{arg}\;\underset{\theta }{\text{min}}\;\text{RSS}(\theta |y)\;,$$

i.e. to minimizing the residual sum of squares. *F *=* g*(*x*(*t*,*u*,*θ*),*θ*) denotes the model response which is in the case of a *state space model* given after integration of a system of differential equations

(3) $$\dot{x}(t)=f(x(t),u(t),\theta )$$

with an externally controlled input function *u* and a mapping to experimentally observable quantities

(4) y(t)=g(x(t),θ)+ε(t).

The parameter vector *θ *comprises the kinetic parameters as well as the initial values, and additional offset or scaling parameters for the observations. Note, that the presented methodology is general, i.e. also applicable for other types of models like regression models or partial differential equations, delay differential equations and differential algebraic equations.

It has been shown [6] that the profile likelihood

(5) $$\text{PL}(\theta_{j})=\underset{\theta_{j\neq i}}{\text{max}}\text{LL}(\theta |y)$$

for a parameter *θ*_*j *_given a data set *y* yields reliable confidence intervals

(6) $$\;{\text{CI}}_{\alpha }(\theta_{j}|y)=\left\{\theta_{j}\;|\;-2\text{PL}(\theta_{j})\leq -2\text{LL}{\left(y\right)}^{*}+\text{icdf}(\chi_{1}^{2},\alpha )\right\}$$

for the estimation of a single parameter. Here, *α *is the confidence level and $\text{icdf}(\chi_{1}^{2},\alpha )$ denotes the *α *quantile of the chi-square distribution with one degree of freedom which is given by the respective inverse cumulative density function. LL^∗^ is the maximum of the log-likelihood function after all parameters are optimized. In (5), the optimization is performed for all parameters except *θ*_*j*_. The analogy of likelihood-based parameter and prediction confidence intervals is discussed in the Additional file 1.

The desired coverage

(7) $$\text{Prob}\left(\theta_{j}\in {\text{CI}}_{\alpha }(\theta_{j})\right)=\alpha \;\;,$$

i.e. the probability that the true parameter value is inside the confidence interval, holds for 6 if the magnitude of the decrease of the residual sum of squares by fitting of *θ*_*j*_ is $\chi_{1}^{2}$ distributed. This is given asymptotically as well as for linear parameters and is a good approximation under weak assumptions [20,21]. If the assumptions are violated, the distribution of the magnitude of the decrease has to be generated empirically, i.e. by Monte-Carlo simulations, as discussed in the Additional file 1.

The *experimental designD *= {*t*,*g*,*u*} comprises all environmental conditions which can be controlled by the experimenter like the measurement times *t*, the observables *g*, and the input functions *u*. A *predictionz *=* F*(*D*_pred_,*θ*) is the response of the model *F* for a prediction condition *D*_pred _= {*t*_pred_,*g*_pred_,*u*_pred_} specifying a prediction observable *g*_pred _evaluated at time point *t*_pred_ given the externally controlled stimulation *u*_pred_. In principle, every quantity which can be computed based on the model can serve as a model prediction *z*. Typical examples comprise concentrations of dynamic compounds, but also concentration ratios or integrals, or characteristics of a time course like the height or width of a peak.

In some cases the observable *g*_pred_ corresponds to measuring a dynamic variable *x*(*t*) directly, i.e. it corresponds to a compound whose concentration dynamics is modeled by the ODEs. In a more general setting the observable is defined by an observational function *g*_pred_(*x*(*t*),*θ*) depending on several dynamic variables *x*. Therefore, *g*_pred _does neither have to coincide with a dynamic variable nor with an observational function *g* of the measurements performed to build the model.

In analogy to (7), the desired property of a prediction confidence interval PCI_*α*_(*D*|*y*) derived from an experimental data set y with a given significance level *α* is that the probability

(8) $$\text{Prob}(F(D_{\text{pred}},\theta_{\text{true}})\in {\text{PCI}}_{\alpha }(D|y))=\alpha$$

that the true value of *F*(*D*_pred_,*θ*_true_) is inside the prediction confidence interval PCI_*α*_is equal to *α*. In other words, the PCI covers the model response for the true parameters with a proportion *α* of the noise realizations which would yield different data sets *y*.

The prediction profile likelihood

(9) $$\text{PPL}(z)=\underset{\theta \in \{\theta |F(D_{\text{pred}},\theta )=z\}}{\text{max}}\;\text{LL}(y|\theta )$$

is obtained by maximization over the model parameters satisfying the constraint that the model response *F*(*Dθ*^∗^) after fitting is equals to the considered value *z* for the prediction. The prediction confidence interval is in analogy to (6) given by

(10) $$\begin{array}{l} {\text{PCI}}_{\alpha }(D_{\text{pred}}|y)=\left\{z\;|\;-2\text{PPL}(z)\leq -2{\text{LL}}^{*}(y)\right)\\ \;\;\;\;+\left(\text{icdf}(\chi_{1}^{2},\alpha )\right\}, \end{array}$$

i.e. the set of predictions *z *=* F*(*D*_pred_*θ*) for which − 2 PPL is below a threshold given by the $\chi_{1}^{2}$-distribution. In analogy to likelihood based confidence intervals for parameters, such PCI yields the smallest unbiased confidence intervals for predictions for given coverage *α*[22].

Instead of sampling a high-dimensional parameter space, the prediction profile likelihood calculation comprises sampling of a one-dimensional prediction space by evaluating several predictions *z*. Evaluating the maximum of the likelihood satisfying the prediction constraint does in general not require an unambiguous point in the parameter space as in the case of structural non-identifiabilities. In analogy to profile likelihood for parameter estimates, the significance level determines the threshold for the PPL, which is given asymptotically by the quantiles (6) of the $\chi_{1}^{2}$-distribution [23]. In the Additional file 1, a Monte-Carlo algorithm is presented which can be used to calculate the threshold in cases where the asymptotic assumption is violated.

### The validation profile likelihood

Likelihood-based confidence interval like (6) or (10) correspond to the set of predictions which are not rejected by a likelihood ratio test. Having a prediction confidence interval, the question arises whether a model has to be rejected if a validation measurement is outside the predicted interval. This, in fact, would hold if a “perfect” validation measurement would be available, i.e. a data point without measurement noise. For validation experiments, however, the outcome is always noisy and is therefore expected to be more frequently outside the PCI than the true value. Hence, the prediction confidence interval (10) has to be generalized for application to a validation experiment.

For a validation experiment, we therefore introduce a *validation profile likelihood *VPL and a corresponding *validation confidence interval*${\text{VCI}}_{\alpha }^{\text{SD}}$ in the following. In such a setting, a confidence interval should have a coverage

(11) $$\text{Prob}\left(z\in {\text{VCI}}_{\alpha }^{\text{SD}}(D_{\text{vali}}|y)\right)=\alpha$$

for the validation data point *z *∼* N*(*μ*,SD^2^) with expectation *μ *=* F*(*D*_vali_,*θ*_true_) and variance SD^2^. Here, *D*_vali_ denotes the design for the validation experiment. A validation confidence interval satisfying (11) allows a rejection of the model if a noisy validation measurement with error SD is outside the interval.

${\text{VCI}}_{\alpha }^{\text{SD}}$ for validation data can be calculated by relaxing the constraint in (9) used to compute the prediction profile likelihood. Because in this case, the model prediction does not necessarily have to coincide with the data point *z*. Instead, the deviation from the validation data point is penalized equivalently to the data *y*. The agreement of the model with the data *y* and the validation measurement *z* is then given by

(12) $$\begin{array}{l} \text{LL}(z,y|\theta )=\underset{=\text{RSS}\;\mathrm{of}\;\text{existing}\;\text{data}\;y}{\underset{︸}{\underset{i}{\sum }{\left(\frac{y_{i}-F(D_{i},\theta )}{\sigma }\right)}^{2}}}\\ \;\;+\underset{=\text{RSS}\;\mathrm{of}\;a\;\mathrm{validation}\;\mathrm{data}\;\mathrm{point}\;z}{\underset{︸}{{\left(\frac{z-F(D_{\text{vali}},\theta )}{\text{SD}}\right)}^{2}}} \end{array}$$

We now define the validation profile (log-)likelihood

(13) $${\text{VPL}}^{\text{SD}}(z|y)={\text{LL}}^{*}(z,y)=\text{LL}(z,y|\theta^{*})$$

with *θ*^∗ ^=* θ*^∗^(*z*,*y*) = arg max_*θ*_ LL(*z*,*y*|*θ*) as the maximized joint log-likelihood in (12) read as a function of *z*. The corresponding validation confidence interval is given by

(14) $$\begin{array}{l} {\text{VCI}}_{\alpha }^{\text{SD}}(D_{\text{vali}}|y)=\left\{z|-2{\text{VPL}}^{\text{SD}}(z|y)\leq -2{\text{LL}}^{*}(z,y)\right)\\ \;\;\;\;+\left(\text{icdf}(\chi_{1}^{2},\alpha )\right\}. \end{array}$$

Optimization of the likelihood (12) minimizes both, the mismatch of existing data RSS(*θ*|*y*), and the mismatch with the fixed validation data point *z*. The model response $F(D_{\text{pred},\theta^{*}})$ obtained after this parameter optimization can be interpreted as a prediction *z*^*″ *^satisfying the constraint optimization problem (9) considered for the prediction profile likelihood. It holds

(15) $$\begin{array}{l} {\text{LL}}^{*}(z,y;\text{SD}>0)-\frac{1}{2}\frac{{\left(z-F(D_{\text{vali}},\theta^{*})\right)}^{2}}{{\text{SD}}^{2}}\\ \;\;\;={\text{LL}}^{*}(z^{\prime \prime },y;\text{SD}=0)\;\;, \end{array}$$

i.e. the validation profile likelihood LL^∗ ^can be scaled to the prediction likelihood via

(16) $$\text{PPL}(z^{\prime }|y)={\text{VPL}}^{\text{SD}}(z|y)-\frac{1}{2}\frac{{\left(z^{\prime }-z\right)}^{2}}{{\text{SD}}^{2}}$$

where *z*^*′ *^=* F*(*D*_vali_,*θ*^∗^(*z*,*y*,SD > 0)) is the model response for *θ*^∗ ^estimated from *z* and *y*.

Optimization with nonlinear constraints is a numerically challenging issue. Therefore, (16) provides a helpful way to omit constraint optimization. The VPL can be calculated with SD > 0 like a common least-squares minimization and is then afterwards rescaled to obtain the PCI for the true value.

## Results

### Small illustration model

First, a small but illustrative model of two consecutive reactions

(17) $$A\;\overset{\theta_{1}}{\to }\;B\;\overset{\theta_{2}}{\to }\;C$$

with rates *θ*_1 _= 0.05,*θ*_2 _= 0.1 and initial conditions *A*(0) =* θ*_3 _= 1,*B*(0) = 0,*C*(0) = 0 is utilized to illustrate our approach. For this purpose, it is assumed that *C*(*t*) is measured at *t *= 0,10,…,100.

For the simulated measurements, Gaussian noise *ε *∼* N*(0,*σ*^2^) with *σ *= 0.1 has been assumed which corresponds to a typical signal-to-noise ratio for applications in cell biology of around 10%. If an experimental setup would not allow for negative measurements, a log-normal distribution of the observational noise could be more appropriate. Then, the Gaussian setting is obtained after a log-transformation of the data [24]. Such transformations and preprocessing procedures would have to be performed before the analysis starts.

**Figure 1:**
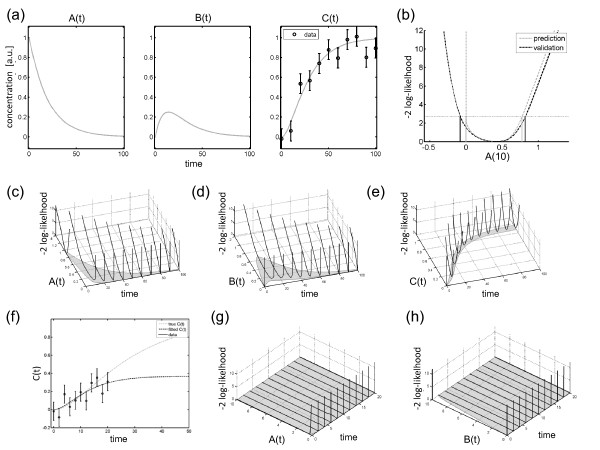
**Illustration model.** The three figures in panel (**a**) show the dynamics and measurement realization for the small model used for illustration purpose. C(t) is measured and the dynamics of all states, i.e. A(t), B(t), and C(t), is intended to be predicted. Panel (**b**) shows as an example the prediction profile likelihood (gray dashed curve) and validation profile likelihood (black dashed curve) of A(t = 10). Thresholding yields confidence intervals for prediction (gray vertical lines) and validation (black vertical lines). The threshold and the respective projections correspond to the *α *= 90% confidence interval. The VCIs are larger than the PCIs, because they account for the measurement error of a validation data point. Panels (**c**)-(**e**) show prediction confidence intervals (gray) for the unobserved states A(t), B(t), as well as for the measured state C(t). The prediction profile likelihood functions are plotted as black curves in vertical direction. Non-observability is illustrated in panels (**f**)-(**h**). Panel (**f**) shows a realization of the measurements for a design which does not provide sufficient information about the steady state of C. This leads to a flat prediction profile likelihood for large values for A(t) as shown in panel (**g**), as well as for B(t) for t > 0 as plotted in panel (**h**). A flat prediction profile likelihood in turn yields unbounded prediction and validation confidence intervals and non-observability of A(t) and B(t) as indicated by the gray shaded regions.

Panel (a) in Figure 1 shows the dynamics of *A*(*t*), *B*(*t*), and *C*(*t*) as well as a typical data realization. This simulated data realization is utilized to calculate the prediction- and validation profile likelihood, e.g. for the dynamic states. Panel (b) shows, as an example, the prediction profile likelihood and the validation profile likelihood for this data realization for predicting *A*(*t*) at time point *t *= 10. The validation profile likelihood has been calculated for validation data with 10% measurement noise, as it was assumed for the measurements. The vertical axis is minus two times the log-likelihood which corresponds to the residual sum of squares RSS. For illustration purposes, the minimum of the log-likelihood LL^∗^ is shifted to zero in all figures. The threshold corresponding to the 90% confidence level is plotted as horizontal line. As explained in the Methods section, the projections to the horizontal axis yields the respective confidence intervals for a prediction or for a validation experiment. The constraint optimization procedure is infeasible for *A*(*t*) ≤ 0 and therefore the PCIs automatically account for strictly positive values of *A*.

The calculation of the prediction and validation confidence intervals has been repeated for *t *= 0,10,…,100 and all three dynamic states *A*(*t*), *B*(*t*), *C*(*t*). In panels (c)-(e), the respective prediction confidence intervals (PCIs) are plotted as well as the prediction profile likelihood. The corresponding validation profile likelihood functions and the respective validation confidence intervals are shown in Additional file 1. Prediction- as well as validation confidence intervals always cover the prediction for the maximum likelihood parameter estimates.

For plotting the confidence intervals along the time axis, the PCIs evaluated the eleven time points have been interconnected by cubic piecewise interpolation. The displayed confidence intervals constitute the propagation of information from the measurements of *C*(*t*) to predictions of the dynamics of the compound concentrations. Because *C* is the measured compound in our example, the prediction confidence intervals for *C* are much smaller than for *A* and *B*. However, also *A* and *B* yield bounded prediction confidence intervals which can be interpreted as *observability* of these dynamic states.

In the Additional file 1, the reliability of our confidence intervals is investigated by calculating the coverage, i.e. by comparing the confidence level with the frequency that the true value is inside the prediction confidence interval. For our example, it is demonstrated that confidence intervals using the asymptotic threshold sometimes yield slightly conservative intervals. Also an algorithm to improve the threshold is provided which yields slightly smaller confidence intervals with the correct coverage.

To illustrate the effect of *non-observability*, the assumption about the available experimental information is slightly changed. The measurements are simulated for earlier and closer time steps, i.e. for *t *= 0,2,…,20. Panel (f) in Figure 1 shows that these time points sample only the transient increase of *C*(*t*). Hence, such a design does not provide sufficient information about the steady state level of *C*. In other words, the modification limits the available information about the total amounts of the compounds, i.e. the concentration dimension of the parameters is practically non-identifiable. This, in turn, manifests in non-observability of the predictions of *A*(*t*) and *B*(*t*).

Panel (g) shows the prediction confidence intervals for *A*(*t*). In the chosen setting, the predictions are unbounded towards infinity and therefore *A*(*t*) is non-observable. In panel (h), it is also shown that *B*(*t*) is non-observable. According to the model definition, *B(0)* is known to be zero, but for *t* > 0, unbounded prediction confidence intervals are obtained which indicate non-observability of *B(t)*.

### MAP kinase signaling model

Next, an ODE model of cellular signal transduction has been used to illustrate our method in a realistic setting. For this purpose, a model of the *mitogen-activated protein (MAP) kinases* which is one of the most extensively studied signal transduction pathway, is utilized. The chosen model [25] consists of eight dynamic states describing the time dependency of the MAP kinases Raf, Raf^∗^, Mek, Mek^∗^, Mek^∗∗^, Erk, Erk^∗^, and Erk^∗∗^which play a very prominent role in many cellular processes, e.g. in cell proliferation. A star ‘*’ denotes phosphorylation of the protein which biologically acts as activation.

**Figure 2:**
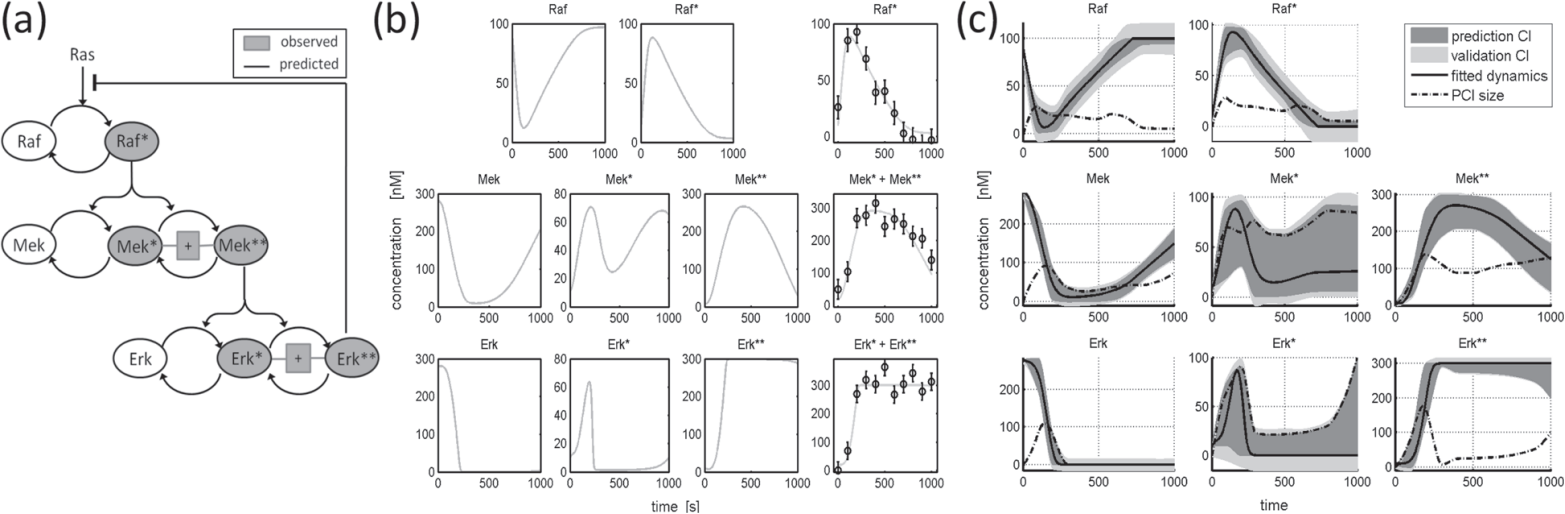
**MAP kinase model.** Panel (**a**) shows the MAP kinase model according to [25]. It is assumed that the phosphorylated compounds are measured. The dynamics of all compounds is intended to be predicted to illustrate the prediction profile likelihood approach. In panel (**b**) the dynamics of the MAP kinase model as well as simulated data set are plotted. The 90% confidence intervals of the dynamic variables for predictions (dark gray) and for validation experiments (light gray) for this noise realization are plotted in panel (**c**). The size of the prediction confidence interval (PCI) is plotted as a dashed-dotted line. In absolute concentrations, the dynamics of Erk^∗∗^has the largest PCI at t = 181 seconds, i.e. when the negative feedback is activated. Also, the dynamics of Mek^∗^is only badly observable in our example. Measurements of both would be very informative for better calibrating the model.

Panel (a) in Figure 2 provides a summary of the MAP kinase signaling pathway. The enzymatic reactions in the ODE model are described as Michaelis-Menten rate equations, i.e. each reaction is parametrized with a maximal enzymatic rate and a Michaelis constant. As in the original publication, the parameters of the two consecutive phosphorylation and dephosphorylation steps of Mek and Erk are assumed to be identical and the initial concentrations are assumed to be known. In this setting, 14 parameters are estimated out of three times eleven data points. Details about the model are provided in Additional file 1.

It is assumed that the total amount of the phosphorylated forms for each protein, i.e. Raf^∗^, the sum of Mek^∗^ and Mek^∗∗^ as well as the sum of Erk^∗^ and Erk^∗∗^, are measured. This observational assumption holds for example for phospho-specific antibodies such as utilized for western blotting. The measurement times are set to 0,100,…,1000 seconds. Again, additive Gaussian noise is assumed. The standard deviation has been set to *σ* = 10 nM.

In panel (b) of Figure 2 a typical noise realization is displayed. Panel (c) shows the prediction confidence intervals (dark gray) and the validation confidence intervals (light gray) for this noise realization calculated for all dynamic states. The size of the confidence intervals is plotted as a dashed-dotted line.

The prediction confidence intervals show how precisely the dynamics is inferred by the available data. The temporal behavior of Raf, Raf^∗^is quite well determined, i.e. the size of the PCI is below 40 nM. Similarly, the unphosphorylated states of Mek and Erk have narrow prediction confidence intervals. For Mek^∗^ the concentration dynamics is only predicted within rather large intervals which for most time points nearly span a range between zero and 100 nM.

The largest absolute size of the prediction confidence interval of 176 nM is obtained for Erk^∗∗^ after 181 seconds. This is the point in time where the negative feedback is activated. Additional experimental investigation of this condition is very informative to further specify the dynamic behavior of the MAP kinase cascade in our example. Further considerations concerning experimental planning are provided in detail in the Additional file 1.

## Discussion

Existing approaches for prediction confidence intervals like MCMC [26] or bootstrap procedures are based on forward evaluations of the model for many parameter values. This works reasonably well for a low dimensional parameter space and if the target density function, i.e. the parameter space to be sampled, is well-behaved [14]. However, sampling nonlinear high-dimensional spaces densely is impractical and it is almost impossible to ensure that sampling the parameter space covers all prediction scenarios. Especially in biological applications the target distributions frequently inherit strong and nonlinear functional relations. In the case of non-identifiability, the parameter space to be sampled is not restricted rendering convergence near to impossible.

In this paper, we present a contrary procedure. The model prediction space is sampled directly and the corresponding model parameters are determined by constraint maximum likelihood to check the agreement of the predictions with the data. This concept yields the prediction profile likelihood which constitutes the propagation of uncertainty from experimental data to predictions.

If a comprehensive prior, i.e. for all parameters, would be available, a Bayesian procedure like MCMC where marginalization, i.e. integration over the nuisance dimensions is feasible could have superior performance. However, in cell biology applications, prior knowledge is very restricted because kinetic rates and concentrations are highly dependent on the cell type and biological context, e.g. on the cellular environment and biochemical state of a cell. Therefore, there is usually at most some prior information for few parameters available. Such prior information can be incorporated in our procedure without restricting its applicability by generalizing maximum likelihood estimation to maximum a-posterior estimation as discussed in the Additional file 1.

In general, generating prediction confidence intervals given the uncertainty in a high-dimensional nonlinear parameter space requires large numerical efforts. However, this complication primarily originates from the complexity of the issue itself rather than from the methodological choice. In fact, the aim is approached by the prediction profile likelihood in a very efficient manner because scanning the parameter space by the constrained optimization procedure to explore the data-consistent predictions is more efficient than sampling parameter space without considering the predictions like it is performed for MCMC. Instead of sampling a high-dimensional parameters space, only the prediction space has to be explored for calculating a prediction profile likelihood, i.e. the optimization of the parameters reduces the high-dimensional sampling problem to exploring a single dimension.

The prediction confidence regions introduced above has to be interpreted *point-wise*. This means that a confidence level *α*controls errors of type 1 which is the probability that the model response for the true parameters is inside the prediction confidence interval for a single prediction condition if many realizations of the experimental data and the corresponding prediction confidence intervals are considered.

In contrast, if a single data set is utilized to generate many prediction intervals, e.g. predictions for several points in time as performed above, the results are *statistically dependent*, i.e. the realization of the PCI of a neighboring time point is very similar and therefore correlated. Therefore, the prediction confidence intervals for a compound for two adjacent points in time very likely both contain the true value, or neither. In such an example, a *common* prediction confidence region for two statistically dependent predictions would require a two-dimensional prediction profile likelihood. This topic, however, is beyond the scope of this article.

The prediction profile likelihood also provides a concept for experimental planning. Experimental conditions with a very narrow prediction confidence interval are very accurately specified by the available data. New measurements for such a condition on the one hand does not provide very much additional information to better calibrate the model parameters, and hence is from this point of view a bad choice for additional measurements. On the other hand, it very precisely predicts the model behavior under these certain conditions and is therefore a very powerful candidate setting for validating the model structure. Contrarily, large prediction confidence intervals indicate conditions which are weakly specified by the existing data and therefore constitute informative experimental designs for better calibrating the model. Because a design optimization on the basis of the prediction profile likelihood does not require any linearity approximation like common experimental design techniques, e.g. based on the *Fisher information*[27], the presented procedure is very valuable for ODE models which are typically highly nonlinear.

Another potential of the prediction profile likelihood shown in this article is its interpretation in terms of *observability*. This term is very commonly used in control theory to characterize whether the dynamics of some unobserved variables can be inferred by the set of feasible experiments. The theory in this field is based on analytical calculations, i.e. the limited amount and inaccuracy of the data is usually not considered. In this article, it has been shown that the prediction profile likelihood allows for a general data-based approach to check whether there is enough information about unobserved dynamic states in the given experimental design and realization of measurements. Therefore, in analogy to the terminology of *practical identifiability*[6], we would suggest to term observability for a given data set, i.e. a restricted prediction confidence interval, as *practical observability*.

Finally, it should be noted, that a prediction could be any function of the compounds and the parameters. In applications, e.g. a ratio of two compound concentrations is a characteristics of interest. In principle also integrals, peak positions and other functions of the dynamic states can be considered as predictions which could be targets for observability considerations as well as for the calculation of prediction and validation confidence intervals. This flexibility renders the prediction profile likelihood as a concept promising to resolve one bottleneck in computer-aided simulations of complex systems, the generation of reliable confidence intervals for predictions.

## Conclusions

Computer-aided simulations are a well-established tool to study a system’s behavior. The applications range from forecasting climate changes [28] via predicting events in a detector in high-energy physics [29] to modeling biological systems [30]. Generating model predictions is a major task in mathematical modeling. For the dynamic mechanistic models as they are applied e.g. in Molecular and Systems Biology, the confidence regions from parameter estimation can have arbitrarily complex shapes. Therefore, it is very difficult or even impossible to sample the parameter space appropriately to generate confidence intervals for predictions. This in turn impedes a data-based observability analysis for the dynamic states.

In this article, the prediction profile likelihood methodology is presented as a method for calculating the set of model predictions which are consistent with existing measurements without explicitly calculating the uncertainty of the parameters. This is performed numerically by constrained optimization and constitutes a powerful tool for assessing model predictions, performing observability analyses, and experimental design. The method is feasible for arbitrary dimensions of the parameter space. It only requires a proper calculation of the maximum likelihood value, i.e. a numerically reliable parameter optimization procedure. The task of sampling a high-dimensional parameter space reduces to scanning a one-dimensional prediction space. It therefore allows the calculation of confidence intervals for model predictions as well as confidence intervals for the outcome of validation experiments.

The applicability of the approach has been shown by a small but instructive system of two consecutive reactions and a published model for MAP kinase signaling. For the small system, it has been shown that the prediction profile likelihood yields desired coverage properties. Moreover, a setting inducing non-observability has been investigated which is characterized by unbounded prediction confidence intervals. For the MAP kinase model, prediction confidence intervals and validation confidence intervals for all dynamic states have been determined on the basis of measurements of the phosphorylated proteins. In addition, the applicability of the approach for experimental planning has been demonstrated.

## Competing interests

The authors declare that they have no competing interests.

## Authors’ contributions

CK developed the method, performed the simulations, and wrote major parts the manuscript. AR contributed to the establishment of the method and wrote parts of the manuscript. JT supported CK and AR in methodological issues and helped to draft the manuscript. All authors read and approved the final manuscript.

## Supplementary Material

Additional file 1 **[Kreutz12_SupplementalMaterial].** In the Supplemental Material, theoretical issues like re-parametrization of the model, coverage, or the accuracy of the asymptotically derived threshold are discussed in detail. Moreover, the computational implementation is described and additional analyses of the two models are provided. Click here for file
