# Supplementary material for: Likelihood based observability analysis and confidence intervals for predictions of dynamic models
Source: BMC Syst Biol. 2012 Sep 5;6:120. doi: 10.1186/1752-0509-6-120 (PMC3490710; doi:10.1186/1752-0509-6-120)
Supplement: Additional file 1 — [Kreutz12_SupplementalMaterial]. In the Supplemental Material, theoretical issues like re-parametrization of the model, coverage, or the accuracy of the asymptotically derived threshold are discussed in detail. Moreover, the computational implementation is described and additional analyses of the two models are provided. [file 1752-0509-6-120-S1.pdf]

Likelihood based observability analysis and confidence intervals for predictions of dynamic models - Supplemental Material -

Clemens Kreutz[1,2*], Andreas Raue[1,7], Jens Timmer[1−6]

[1] Physics Department, University of Freiburg, Hermann Herder Straße 3, 79104 Freiburg, Germany,
[2] Freiburg Centre for Biosystems Analysis (ZBSA), University of Freiburg, Habsburgerstraße 49, 79104 Freiburg, Germany,
[3] Freiburg Institute for Advanced Studies (FRIAS), University of Freiburg, Albertstraße 19, 79104 Freiburg, Germany,
[4] Freiburg Initiative in Systems Biology (FRISYS), University of Freiburg, Schaenzlestraße 1, 79104 Freiburg, Germany,
[5] BIOSS Centre for Biological Signalling Studies, University of Freiburg, Schaenzlestraße 18, 79104 Freiburg, Germany,
[6] Department of Clinical and Experimental Medicine, Universitetssjukhuset, 58183 Linköping, Sweden,
[7] Institute of Bioinformatics and Systems Biology, Helmholtz Zentrum München, Ingolstädter Landstraße 1, 85764 Neuherberg, Germany

Email: CK: ckreutz@fdm.uni-freiburg.de; AR: andreas.raue@fdm.uni-freiburg.de; JT: jeti@fdm.uni-freiburg.de;

*Corresponding author

As Supplementary Material, the relationship between parameter- and prediction confidence intervals is discussed in section 1. It is justified that for prediction confidence intervals the asymptotic threshold is given by the chi-square distribution. Analytical calculations of prediction confidence intervals are discussed in section 2.

In section 3, a Monte-Carlo approach is presented which can be utilized to check the asymptotic threshold in non-asymptotic settings. For our example, it is shown that the threshold according to the asymptotic setting is slightly too large for the illustration model. As a consequence, the predictions confidence intervals are conservative, as shown in section 4. However, in terms of false positives, conservative confidence intervals are not a serious limitation. It is also argued that the Monte-Carlo approach presented in section 3 can be utilized to calculate exact thresholds for non-asymptotic settings. In section 4 it is proven, that these thresholds yield a correct coverage.

Both, the size of the prediction confidence interval and the magnitude of the measurement error of a validation data point have an impact on the size of a validation confidence interval. In an asymptotic setting the contributions of both is closely related to the formula $Var(F + Z) = Var(F) + Var(Z)$ for the variance of the sum of two independent random variables. Here, F denotes the random variable corresponding to predicted value, and Z the random variable for validation measurement. The relationship between the

magnitudes of the prediction- and of the validation confidence intervals is discussed in more detail section 5.

For a Bayesian setting, i.e. if prior probabilities for parameters are intended to be utilized in an analysis, the methodology presented in the main text which is based on maximum likelihood estimation can be generalized to maximum a-posteriori estimation. This aspect is discussed in section 6. For illustration purpose, prediction confidence intervals using a log-normal prior for a single parameter are calculated for the example model consisting of two consecutive reactions.

In addition to the prediction confidence intervals shown in Fig. 1 in the main text, validation confidence intervals are shown in section 7. A further example for an observability analysis is presented in section 8.

Details about the MAP kinase model investigated in the main text, are provided in section 9. Experimental design considerations based on the prediction profile likelihood are discussed in section 10. Details about the numerical implementation are given in section 11.

1 Re-parametrization

Parameter estimation, i.e. the prediction of a parameter value out of experimental data, can be seen as a special case of a model prediction. Then, the parameter profile likelihood coincides with the prediction profile likelihood and the respective parameter confidence intervals correspond to prediction confidence intervals. In this sense, the prediction profile likelihood generalizes the parameter profile likelihood. In fact, the idea of the prediction profile likelihood and the calculation of prediction confidence intervals, e.g. the choice of the threshold, is very intuitive for this special case.

In other situations, i.e. if the prediction does not coincide with a parameter, an analog strategy would require a re-parametrization of the model in a way that the desired model prediction is unambiguously given by the value of a single new parameter. Then, again the profile likelihood for the new parameter would give a confidence interval for the prediction. In this case, without loss of generality such a parameter can be denoted by θ'_1. Then, the re-parametrization would be a transformation

$$T : \{\theta_1, \ldots, \theta_{n_p}\} \to \{\theta'_1, \ldots, \theta'_{n_p}\} \tag{1}$$

of the n_p parameters θ to new parameters $\theta'_1, \ldots, \theta'_{n_p}$ where all predictions for the condition $D_{\rm pred}$ satisfy

$$F'(D_{\rm pred}, \theta') = F'(D_{\rm pred}, \theta'_1) \ . \tag{2}$$

Here, $F' = F \circ T^{-1}$ denotes the model for the transformed parameters. For a transformation satisfying (2), any change of the parameters $\theta'_2, \ldots, \theta'_{n_p}$ would not affect F', because T is chosen in a way that the effect of $\theta_2, \ldots, \theta_{n_\theta}$ is orthogonal to the effects of θ_1.

However, because ODE systems can only be solved analytically for special cases, such a re-parametrization cannot be found explicitly for most realistic models. This restriction can be resolved numerically by an implicit re-parametrization which is obtained by a constrained nonlinear optimization procedure. This idea yields the prediction profile likelihood

$$\mathrm{PPL}(z) = \max_{\theta \in \{\theta | F(D_{\mathrm{pred}}, \theta) = z\}} \mathrm{LL}(y|\theta) \tag{3}$$

which is obtained by maximization over the model parameters satisfying the constraint that the model response $F(D, \theta^*)$ after fitting is equals to the considered value z for the prediction. In this case, the 'new parameter' is the predicted value itself, i.e. $z \equiv \theta'_1$ and F' is the identity function.

Equation (3) also resolves the formal issue which occurs if there is not a unique parameter set θ given by the constraint $F(D_{\mathrm{pred}}, \theta) = z$. If there are several such parameter sets, the ambiguities either vanish by taking the parameter set with maximize the log-likelihood, or they are not relevant because only the value of the maximized log-likelihood enters the calculation.

2 Analytically derived prediction confidence intervals

Analytically derived confidence intervals for model predictions are usually calculated in an approximative manner by Taylor expansion

$$\mathrm{LL}(y|\theta) \approx \mathrm{LL}(y|\hat{\theta}) + \underbrace{\frac{\partial}{\partial \theta} \mathrm{LL}(y|\hat{\theta})}_{=0 \text{ for } \hat{\theta} = \arg\max_\theta \mathrm{LL}(y|\theta)} \left(\theta - \hat{\theta}\right) + \frac{1}{2} \frac{\partial^2}{\partial \theta^2} \mathrm{LL}(y|\hat{\theta}) \left(\theta - \hat{\theta}\right)^2 \tag{4}$$

of the log-likelihood up to the second order around the maximum likelihood estimate. This approach yields asymptotic confidence intervals which can be calculated for ODE models.

In contrast to asymptotic or numerically calculated prediction confidence intervals, an exact analytical calculation in general involves the following steps.

1. An explicit analytical expression for the model observables is required, i.e. $g(x(t), \theta)$, for all measured conditions. For state space models, this comprises an analytical solution $x(t, \theta)$ of the ODEs.
2. The likelihood or the log-likelihood $\mathrm{LL}(y|\theta)$ has to be written in an explicit manner.
3. The maximum likelihood estimate $\arg\max_\theta \mathrm{LL}(y|\theta)$ is calculated by $\frac{\partial \mathrm{LL}}{\partial \theta} = 0$ and resolving for θ.
4. The parameter confidence region $CI_\alpha(\theta|y) = \left\{\theta \mid LL(y|\theta) \leq LL(y|\hat{\theta}) + \mathrm{icdf}(\chi^2_1, \alpha)\right\}$, has to be calculated for the significance level α.

5. The desired model prediction $z(\theta)$ has to be stated in an explicit analytical manner.

6. The parameter confidence region $CI_\alpha(\theta|y)$ is translated into prediction confidence region $\{z(\theta) \mid \theta \in CI_\alpha(\theta|y)\}$

Let's assume as an illustrating show case a dynamics given by

$$\dot{x} = -\theta \tag{5}$$

and given $x(0) = 1$. For simplicity a single data point $y = 0.9$ for x at time point $t = 1$ is assumed in addition as well as Gaussian measurement noise $N(0, 0.1^2)$. The solution of the ODE is for this example given by $x(t) = \exp(-\theta t)$ and the observational function reads $g(t = 1) = \exp(-\theta)$ for the data point. The log-likelihood is

$$\text{LL}(y|\theta) = \log\left[\frac{1}{\sqrt{2\pi\sigma^2}} \exp\left(-\frac{(0.9 - \exp(-\theta))^2}{2\,\sigma^2}\right)\right] = -50\,(0.9 - \exp(-\theta))^2 + c \tag{6}$$

with $c = 1/\sqrt{2\,\pi\sigma^2}$ and the maximum likelihood estimate is obtained by

$$\begin{aligned} \frac{\partial}{\partial\theta}\text{LL}(y|\theta) &= 0 & (7)\\ \Leftrightarrow -50\,\theta\,(0.9 - \exp(-\theta)) &= 0 & (8)\\ \Rightarrow \hat{\theta} &= -\log(0.9) = 0.1054\ . & (9) \end{aligned}$$

Let's assume as an example, the desired prediction is given by $z = \theta^2$. The prediction confidence interval is defined as

$$\left\{z(\theta) \mid \text{LL}(y|\theta) \leq \text{LL}(y|\hat{\theta}) + \text{icdf}(\chi^2_1, \alpha)\right\}\ . \tag{10}$$

For $\alpha = 0.95$, the threshold is $\text{icdf}(\chi^2_1, \alpha) = 3.8415$. For the boundaries of the parameter confidence interval it holds

$$\begin{aligned} \text{LL}(y|\theta) &= \text{LL}(y|\hat{\theta}) + \text{icdf}(\chi^2_1, \alpha) & (11)\\ \Leftrightarrow 50\,(0.9 - \exp(-\theta))^2 + c &= c + 3.8415 & (12)\\ \Rightarrow \theta = -0.1631 &\wedge \quad \theta = 0.4734 & (13) \end{aligned}$$

and therefore the parameter confidence interval is $\theta \in [-0.1631, 0.4734]$. The prediction confidence interval for $z = \theta^2$ is given by $z \in [0, 0.2241]$. Note that because the observation as well as the prediction are non-linear with respect to the parameter θ, both, the parameter confidence interval as well as the prediction

confidence interval are asymmetric around $\hat{\theta} = 0.1054$, i.e. around $z(\hat{\theta}) = 0.0111$, respectively. In contrast, asymptotic confidence intervals according to (4) would erroneously yield symmetric intervals.

For the example $A \rightarrow B \rightarrow C$ discussed in the main text, the ODEs are linear and therefore analytical integration of the ODEs is feasible yielding

$$A(t) = A(0)\, e^{-\theta_1 t} \tag{14}$$

$$B(t) = A(0)\, \frac{\theta_1}{\theta_2 - \theta_1} \left(e^{-\theta_1 t} - e^{-\theta_2 t}\right) \tag{15}$$

$$C(t) = A(0) \left(1 - \frac{\theta_2 e^{-\theta_1 t} - \theta_1 e^{-\theta_2 t}}{\theta_2 - \theta_1}\right) . \tag{16}$$

For given measurements y of $C(t)$ for $t = 0, 10, \ldots, 100$, the log-likelihood is

$$\mathrm{LL}(y|\theta) = \sum_{i=1}^{11} \frac{(y_i - C(t_i))^2}{2\sigma^2} + const. \tag{17}$$

and the maximum likelihood estimates are given by

$$\frac{\partial \mathrm{LL}\ (y|\theta)}{\partial \theta_1} = 0 \tag{18}$$

$$\frac{\partial \mathrm{LL}(y|\theta)}{\partial \theta_2} = 0 \tag{19}$$

$$\frac{\partial \mathrm{LL}(y|\theta)}{\partial \theta_3} = 0 \tag{20}$$

which, however, cannot be resolved analytically with respect to the parameters. Therefore, even for this easy example, there is no explicit formula for the maximum likelihood estimate and parameter- as well as prediction confidence intervals have to be calculated numerically, i.e. like presented in this manuscript, or in an approximate manner.

3 Profile likelihood threshold

A suitable parameter transformation makes the prediction profile likelihood equivalent to the parameter profile likelihood. Therefore, the following discussion holds for both, for parameter and for prediction confidence intervals.

In general, fitting a model to experimental data reduces the residual sum of squares RSS. In the asymptotic case, i.e. for a large number of data points, it can be shown that the decrease of RSS due to fitting one parameter is chi-square distributed with one degree of freedom. This result also holds exactly in the non-asymptotic case for linear parameters. This outcome is utilized to define the asymptotic threshold for profile likelihood confidence intervals [1].

```
Fit the model M to the measurements obtaind for the design D.
This model is considered as the 'truth'.

Define the prediction design D_pred.
Evaluate true model prediction z_true = F(D_pred).

for a noise realization ε_i
     y_i(D) = SIMULATE_DATA (M, D, ε_i)
     PPL*_i = CALIBRATE_MODEL (y_i(D), M)
     PPL_i(z_true) = CALIBRATE_MODEL_CONSTRAINT (y_i(D), M, z_true)
     epdf_i = PPL_i(z_true) − LL*_i
 threshold(α) = quantile_α(ecdf)
```

Figure 1: A Monte-Carlo algorithm for calculating the profile likelihood threshold empirically. In general, new noise realizations $y_i(D)$ are utilized to calculate the distribution of $\mathrm{PPL}_i(z_{\mathrm{true}}) - \mathrm{LL}_i^*$. The α quantile of this distribution can be used as a threshold for prediction confidence intervals instead of the asymptotic threshold, i.e. instead of the α quantile of the χ_1^2 distribution.

For nonlinear parameters, the distribution of the decrease of the residual sum of squares by the parameter estimation procedure, has not yet been derived for the general setting. However, since the profile likelihood based confidence intervals are independent on bijective transformations of the parameter space [2], the assumption also holds if there is such a transformation, which makes the parameter of interest linear at least within its confidence interval. Such a transformation only has to exist, it is not required to derive it analytically.

A situation where such a transformation does not exist occurs if the nonlinearity yields a non-monotone dependency of the profile likelihood, i.e. there are several local minima in the confidence interval. In this case, there is a larger decrease of the residual sum of squares and the standard threshold yields conservative results, i.e. the calculated confidence intervals are too large for the desired confidence level α.

In Fig. 1, a procedure is presented for checking the standard threshold. It is a Monte-Carlo analysis of the impact of the nonlinear constraint used to calculate the prediction profile likelihood on the magnitude of overfitting.

In Fig. 2, the asymptotic thresholds corresponding to $\alpha = 0.05, 0.1, \ldots, 0.95, 0.99$, i.e. the quantiles of the χ_1^2 distribution, are compared with the empirically calculated thresholds for several prediction scenarios. Here, 1000 realizations of simulated data have been evaluated. The error bars plotted in this figure are bootstrap confidence intervals of the quantiles which have been determined out of 10^4 bootstrap samples.

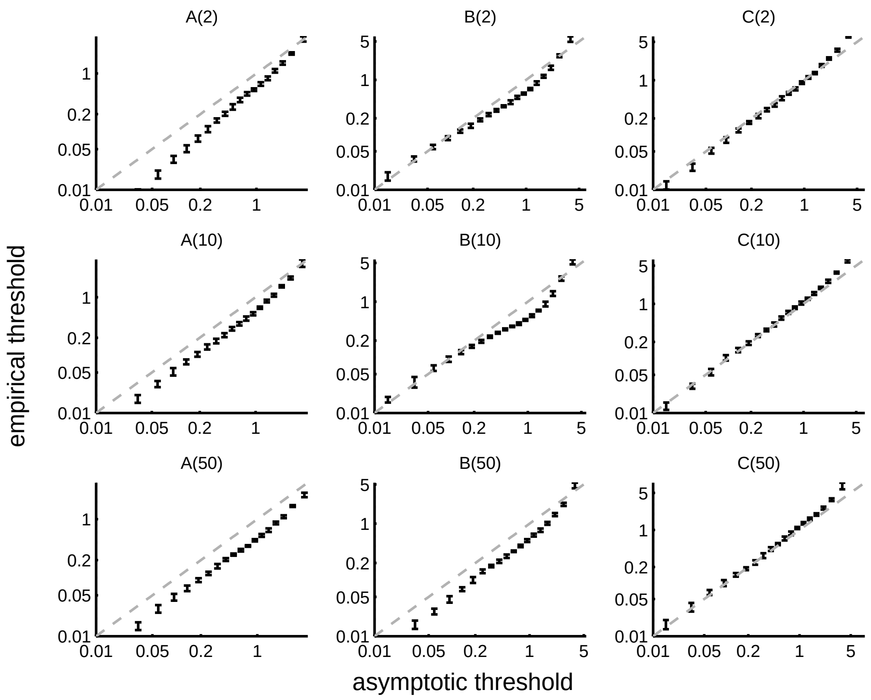

Figure 2: The Monte-Carlo approach allows a comparison of the asymptotic thresholds with the empirically calculated, i.e. the correct thresholds. Here, the thresholds corresponding to $0.05, 0.1, \ldots, 0.95, 0.99$ confidence levels have been plotted for nine different prediction scenarios for the model $A \rightarrow B \rightarrow C$. In our example, the asymptotic thresholds are slightly too large for predictions of $A(t)$ and $B(t)$ which makes the asymptotic confidence intervals conservative.

For every bootstrap sample, 1000 runs are drawn with replacement from the original 1000 runs and the α-quantile of $\mathrm{PPL}(z_{\mathrm{true}}) - \mathrm{LL}^*(y)$ is evaluated to generate the empirical distribution of the correct threshold for α confidence intervals.

For the model

$$A \rightarrow B \rightarrow C \tag{21}$$

the asymptotic thresholds are slightly too large for predicting $A(t)$ and $B(t)$ on the basis of measurements of $C(t)$. This makes the asymptotic confidence intervals conservative in this example. In general, the non-linearity of ODE models can yield conservative as well as anti-conservative thresholds, although we observed only conservative situations in applications so far. Providing a comprehensive theoretical investigation of the conditions yielding conservative asymptotic thresholds is beyond the scope of this article and is discussed in more detail in [3]. The impact on the coverage is discussed in the following section.

4 Coverage

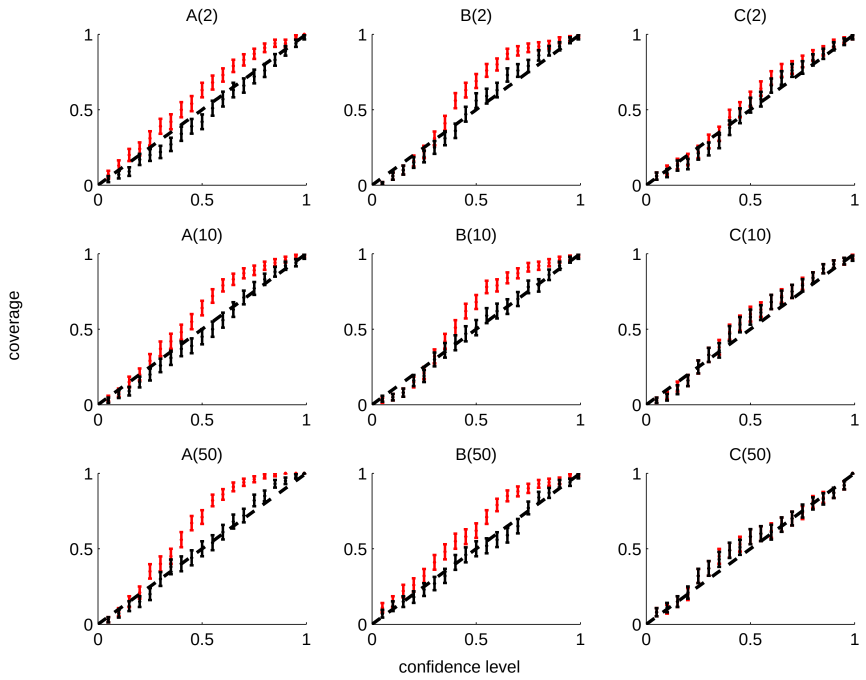

Figure 3: Coverage of the prediction confidence intervals for the consecutive model. The horizontal axis is the confidence level $\alpha = \{0.05, 0.1, \ldots, 0.95, 0.99\}$ which constitutes the desired coverage of the confidence intervals. The vertical axis is the realized coverage obtained for hundred data realizations. The red error bars are the result obtained for the asymptotic threshold. Because the coverage is larger than the confidence levels for some scenarios, asymptotic thresholds yield conservative outcomes for predictions of $A(t)$ and $B(t)$. The black error bars indicate the results for the Monte-Carlo thresholds which shows almost perfect agreement with the confidence level in all prediction scenarios.

The coverage

$$C = \text{Prob}(F(D_{\text{pred}}, \theta_{\text{true}}) \in \text{PCI}_\alpha(D|y)) \quad (22)$$

is the probability that the $\text{PCI}_\alpha(z|y)$ contains the true value $F(D_{\text{pred}}, \theta_{\text{true}})$. A desired property of any confidence interval is that the coverage coincides with the confidence level α.

Fig. 3 shows the estimated coverage of the prediction confidence intervals calculated for nine different prediction scenarios. In these scenarios $A(2)$, $B(2)$, $C(2)$, $A(10)$, $B(10)$, $C(10)$, $A(50)$, $B(50)$, $C(50)$ have been predicted, i.e. all three dynamic variables are predicted for an early, an intermediate, and a late point in time. For this analysis, a hundred runs with different noise realizations have been analyzed. The error bars plotted in this figure are bootstrap confidence intervals of the mean coverage which have been determined out of 10^4 bootstrap samples. For every bootstrap sample, hundred runs are drawn with replacement from the

original runs and the coverage is evaluated to generate the empirical distribution of the coverage estimates. Increasing the number of runs would decrease these error bars but would not change the expectation of the coverage estimates.

The coverage obtained for the asymptotic threshold (red) tends to be conservative, i.e. the true model response is inside the confidence interval more frequently as specified by the confidence level α. This means that there are more false negatives than intended which does not constitute a serious problem in terms of validity of conclusions. In contrast, an anti-conservative coverage would constitute an issue because an increased false positive rate could lead to invalid reasoning. Because a conservative threshold yields an increased coverage, the results obtained in this section are closely related to the discussion in the previous section 3.

The coverage obtained by the adjusted thresholds obtained by the Monte-Carlo algorithm shown in Fig. 1 are displayed by the black error bars in Fig. 3. Here, the coverage coincides with the confidence level which confirms the validity of the prediction profile likelihood based confidence intervals.

5 Comparison of PCI and VCI

For Gaussian noise, the validation profile likelihood satisfies

$$\mathrm{VPL}^{\mathrm{SD}}(z|y) \leq \mathrm{PPL}(z_{\mathrm{alt}}|y) + \frac{1}{2}\frac{(z_{\mathrm{alt}} - z)^2}{\mathrm{SD}^2} \;\;, \forall z_{\mathrm{alt}} \tag{23}$$

for any alternatively predicted value z_{alt}. On the one hand, the inequality can be utilized to interpret a difference between the respective confidence intervals. Furthermore, the equation can be utilized for consistency checks, e.g. to prove the numerically calculated VPL and PPL. Small differences between the size of the $\mathrm{VCI}^{\mathrm{SD}}$ and PCI indicate a flat prediction profile likelihood close to the threshold whereas deviations of the confidence intervals in the order of magnitude of SD occur if the PPL has a large slope. This aspect is illustrated in the following.

For illustration purpose, an asymptotic setting with a quadratic prediction profile likelihood

$$-2\,\mathrm{PPL}(z) = \frac{z^2}{\mathrm{SE}^2} \tag{24}$$

with $\mathrm{SE} \in \{0.1, 0.5, 1, 2\}$ has been assumed. These four settings are shown in Fig. 4. The prediction profile likelihood is shown as a red line. For several z_{alt}, the quadratic term in (23) is plotted by blue curves attached to the PPL. The VPL constitutes the infimum of these curves which in this special case can be calculated

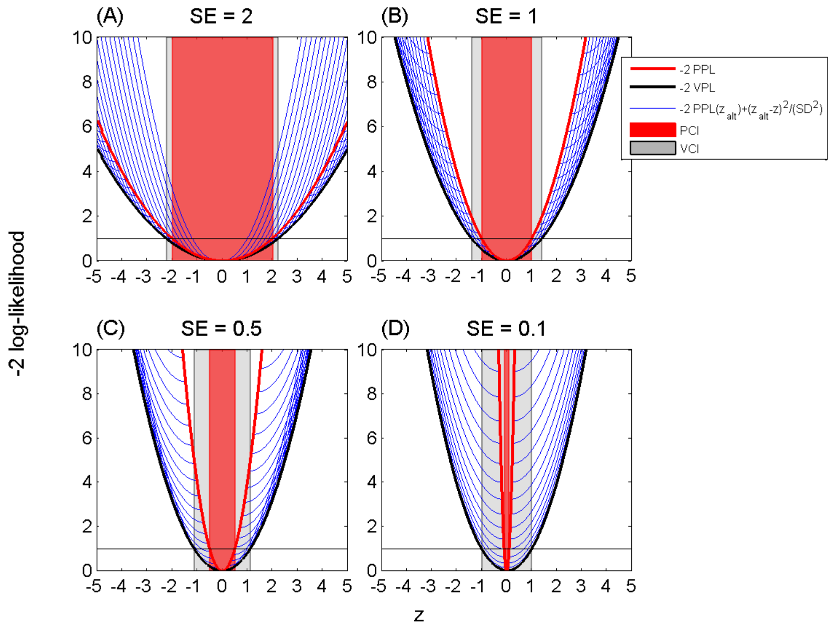

Figure 4: Comparison of prediction and validation confidence intervals. Panel (A) shows a prediction profile likelihood (red line) with a rather flat shape. Here, the curvature of the prediction profile likelihood corresponds to a prediction standard error SE = 2. In this case, the prediction confidence intervals are large (red shaded) and the increase of the validation confidence intervals (gray) is smaller than indicated by the validation data error SD. If the data is more informative, i.e. SE decreases (panels B-D), the slope of prediction profile likelihood increases yielding larger difference between the PCI and VCI.

analytically and is given by

$$-2\,\mathrm{VPL}^{\mathrm{SD}}(z) = \frac{z^2\mathrm{SE}^2}{\left(\mathrm{SD}^2+\mathrm{SE}^2\right)^2} + \left(\frac{z}{\mathrm{SD}} - \frac{z\,\mathrm{SE}^2}{\mathrm{SD}\left(\mathrm{SD}^2+\mathrm{SE}^2\right)}\right)^2 . \tag{25}$$

Panel (A) shows the comparison for SE > SD. In this case, the boundaries of the VCI and the PCI differ only by a value around 0.38. In Panel (B), SE is chosen equal to SD. In Panels (C) and (D) SE is further decreased. This corresponds to more informative data for predicting the exact value of z. In these cases, the optimum of the PPL is narrow in comparison to validation data error SD. Then, during fitting the model, a mismatch $z - z^*$ is predominantly explained by the observation error of the validation data point. The difference of the boundaries of the confidence intervals increase and approach the 1-(1-0.6827)/2 = 84.14% quantile of the Gaussian distribution, i.e. a value $\mathrm{icdf}(N(0, \mathrm{SD}=1), .8414) = 1$ which is the two-sided 68% confidence interval for a validation data point for a constant model prediction, i.e. for $\mathrm{SE} \to 0$.

In an asymptotic setting, the predicted values as well as the validation data point are normally distributed and the size of confidence intervals for any α is given by the respective variances. If the predicted value is

denoted by a random variable F with variance $Var(F)$ and the validation data point by a random variable Z with variance $Var(Z)$, the general formula for the variance

$$Var(F, Z) = Var(F) + Var(Z) \tag{26}$$

of the sum of two independent random variables can be utilized to calculate the size

$$|\mathrm{VCI}| = \sqrt{Var(F) + Var(Z)} = \sqrt{\mathrm{SE}^2 + \mathrm{SD}^2} \tag{27}$$

of the 68% validation confidence interval. Because variances, i.e. the squared standard errors and standard deviations, behave additively, the largest value of SD and SE dominates the size of the VCI. From the inequalities $Var(F + Z) > Var(F)$ and $Var(F + Z) > Var(Z)$, it follows, as an example, that a lower boundary of the size of the 68% validation confidence interval is given by the maximum

$$|\mathrm{VCI}| \geq \max(\mathrm{SD}, \mathrm{SE}) \tag{28}$$

of the 68% prediction confidence interval and the standard deviation of the measurement error for the validation data point.

6 Prior information

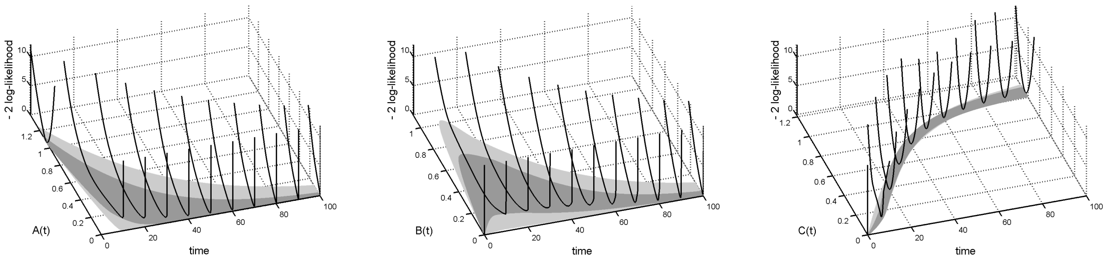

Figure 5: The curves in vertical direction are the prediction profile likelihood functions for $A(t)$ (left panel), $B(t)$ (middle), and $C(t)$ (right panel) if a log-normal prior for θ_3 is assumed. The respective 90% confidence intervals are plotted in dark gray. The light gray regions indicate the 90% confidence intervals if the parameter θ_3 is estimated without prior information.

If prior information about parameters is available, e.g. a prior distribution $\pi(\theta)$, maximum likelihood estimation is replaced by maximum a-posteriori (MAP) estimation

$$\hat{\theta}_{\mathrm{MAP}} = \arg\max_{\theta} \rho(y|\theta)\pi(\theta) \tag{29}$$

$$= \arg\max_{\theta} \left(\mathrm{LL}(y|\theta) + \log(\pi(\theta))\right) \ , \tag{30}$$

i.e. the parameters are estimated by maximizing the a-posterior probability of the data and the parameter estimates. For most common priors, MAP estimation can be performed by MLE using a penalized likelihood. As an example, a log-normal prior for a parameter component θ' yields

$$\mathrm{LL}_{\mathrm{prior}} = \mathrm{LL} - \frac{1}{2}\frac{(\log(\theta') - \langle\theta'\rangle)^2}{\mathrm{Var}(\theta')} + const \ . \tag{31}$$

To incorporate prior knowledge, the presented prediction profile likelihood approach has to be generalized to MAP estimation and the penalized likelihood (31) is used instead of the standard log-likelihood LL.

To illustrate the incorporation of prior knowledge for parameter values, the initial concentration $A(0) = \theta_3$ is assumed to be drawn from a log-normal distribution

$$\theta_3 \sim logN(0, 1) \tag{32}$$

with expectation $\langle\log(\theta_3)\rangle = 0$ and variance $Var(\log(\theta_3)) = 0.1$. For parameter estimation, this is accounted for by using the penalized likelihood (31), i.e. by adding an additional term to the residual sum of squares.

As in the example in the main text, the calculation of the prediction and validation confidence intervals has been repeated for $t = 0, 10, \ldots, 100$ and all three dynamic states $A(t)$, $B(t)$, $C(t)$. In this example, the true value of $A(0) \equiv \theta_3$ has been drawn according to the prior from the log-normal distribution (32).

Fig. 5 shows the prediction profile likelihood functions as curves in vertical direction as well as the respective 90% prediction confidence intervals as dark gray shaded regions. The prediction confidence intervals plotted in light color are obtained if θ_3 is estimated without prior information. Because C is the measured compound in our example, the prediction confidence intervals for C are much smaller than for A and B. However, also A and B yield bounded prediction confidence intervals which can be interpreted as *observability* of these dynamic states. Omitting the prior information yields larger prediction confidence intervals, especially for the unobserved states $A(t)$ and $B(t)$.

7 Validation profile likelihood for the consecutive reaction model

In the main text, prediction confidence intervals have been shown for the consecutive reaction model. Fig. 6 shows the corresponding validation profile and the respective validation confidence intervals for the same noise realization for all dynamic variables $A(t)$, $B(t)$, and $C(t)$. Validation confidence intervals account for the measurement noise in a validation experiment. Therefore, they are larger than the prediction confidence intervals shown in the main text in Fig. 1, panels (c)-(e).

Because Gaussian noise $\varepsilon \sim N(\mu, SD^2)$ has been assumed, the validation confidence intervals covers negative values if the true model response $\mu = F(D_{\mathrm{pred}}, \theta_{\mathrm{true}})$ is close to zero.

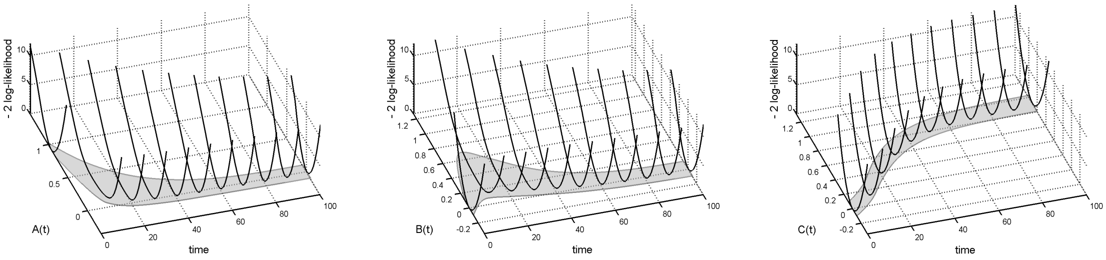

Figure 6: The left panel shows the validation confidence intervals for the unobserved state $A(t)$. The validation profile likelihood functions are plotted as curves in vertical direction. For the plotting the confidence intervals along the time axis, the VCIs have been interconnected by cubic piecewise interpolation. Validation confidence intervals and validation profile likelihood functions for the intermediate unobserved state $B(t)$ are shown in the middle and for $C(t)$ in the right panel.

8 Observability of the long-term dynamics

In the main text, it has been discussed how to exploit the prediction profile likelihood for observability analyses. For the two step model

$$A \overset{\theta_1}{\rightarrow} B \overset{\theta_2}{\rightarrow} C \tag{33}$$

it has been shown that measurements of the compound C which sample only the transient increase and therefore does not provide information about the steady state level lead to non-observability of $A(t)$, and $B(t)$ for $t > 0$. In addition to that result, Fig. 7 shows prediction confidence intervals for $C(t)$ for times much larger than the measurement times $t = 0, 2, \ldots, 20$. $C(t)$ becomes practically non-observable for times which are much larger than the time sampling interval.

9 Characteristics of the MAP kinase model

To demonstrate the applicability of our approach in a realistic setting, the published model of MAP kinase signaling [4] has been utilized in the main text to illustrate the calculation of prediction and validation confidence intervals.

Fig. 8 shows the long-term dynamics of this model, i.e. the oscillations. In our analysis only the initial phase, i.e. the first 1000 seconds have been considered. This time interval is characterized by strong nonlinearity of the model response with respect to the parameters and constitutes a compromise setting between a transient and an oscillatory dynamics.

Tab. 1 summarizes the model parameters as they have been published in [4]. The Hill coefficient n is assumed to be equal to one. For the observational noise of the validation data, the same noise level as for

Symbol	Description	Value	Lower boundary	Upper boundary	Units
V_1	max. enzyme rate	2.5	1e-8	1e6	nM s^{-1}
n	Hill coefficient of the feedback	1	1	1	1
K_I	Michaelis constant	9	1e-8	1e6	nM
K_1	Michaelis constant	10	1e-8	1e6	nM
V_2	max. enzyme rate	0.25	1e-8	1e6	nM s^{-1}
K_2	Michaelis constant	8	1e-8	1e6	nM
k_3	catalytic rate constant	0.025	1e-8	1e6	nM s^{-1}
K_3	Michaelis constant	15	1e-8	1e6	nM
k_4	catalytic rate constant	0.025	1e-8	1e6	nM s^{-1}
K_4	Michaelis constant	15	1e-8	1e6	nM
V_5	max. enzyme rate	0.75	1e-8	1e6	nM s^{-1}
K_5	Michaelis constant	15	1e-8	1e6	nM
V_6	max. enzyme rate	0.75	1e-8	1e6	nM s^{-1}
K_6	Michaelis constant	15	1e-8	1e6	nM
k_7	catalytic rate constant	0.025	1e-8	1e6	nM s^{-1}
K_7	Michaelis constant	15	1e-8	1e6	nM
k_8	catalytic rate constant	0.025	1e-8	1e6	nM s^{-1}
K_8	Michaelis constant	15	1e-8	1e6	nM
V_9	max. enzyme rate	0.5	1e-8	1e6	nM s^{-1}
K_9	Michaelis constant	15	1e-8	1e6	nM
V_{10}	max. enzyme rate	0.5	1e-8	1e6	nM s^{-1}
K_{10}	max. enzyme rate	15	1e-8	1e6	nM

Table 1: Parameter values of the MAP kinase model as published in [4]. For numerical stability, the admissible parameter range has been restricted.

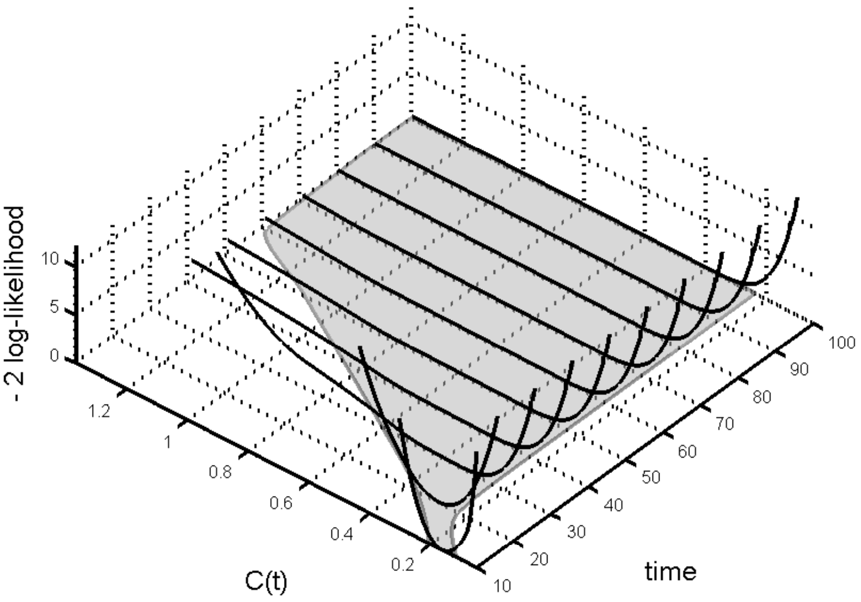

Figure 7: Prediction confidence intervals for the extrapolation of $C(t)$ to time points much larger than the measurement times. Because in this example the experimental design does not provide sufficient information about the steady state level of $C(t)$, the prediction confidence intervals diverge and the steady state $C(t)$ for $t \to \infty$ is non-observable.

the experimental data has been assumed, i.e. $\sigma = \mathrm{SD} = 10$.

10 Experimental design conclusions

The size of prediction confidence interval can be utilized to figure out informative experimental designs. If the information of a single data points is intended to be evaluated, then the validation confidence intervals are appropriate. If many experimental replicates are feasible, the average observation will have a small standard error and then prediction confidence intervals can be used to assess a design.

Fig. 9 shows the size of the 90% prediction confidence intervals (upper row), i.e. the difference between the upper and lower boundary, and the size of the validation confidence intervals (lower row) along the time axis. The size is plotted in absolute concentrations (left panels) and relative to the total amount of the protein (right panels).

Independently from the way of the assessment, Erk (blue lines) yields the smallest prediction and validation confidence intervals for $300 < t \leq 1000$. Therefore, measurements of Erk in this time interval constitute very informative experimental designs for testing the model. As discussed in the main text, such a setting

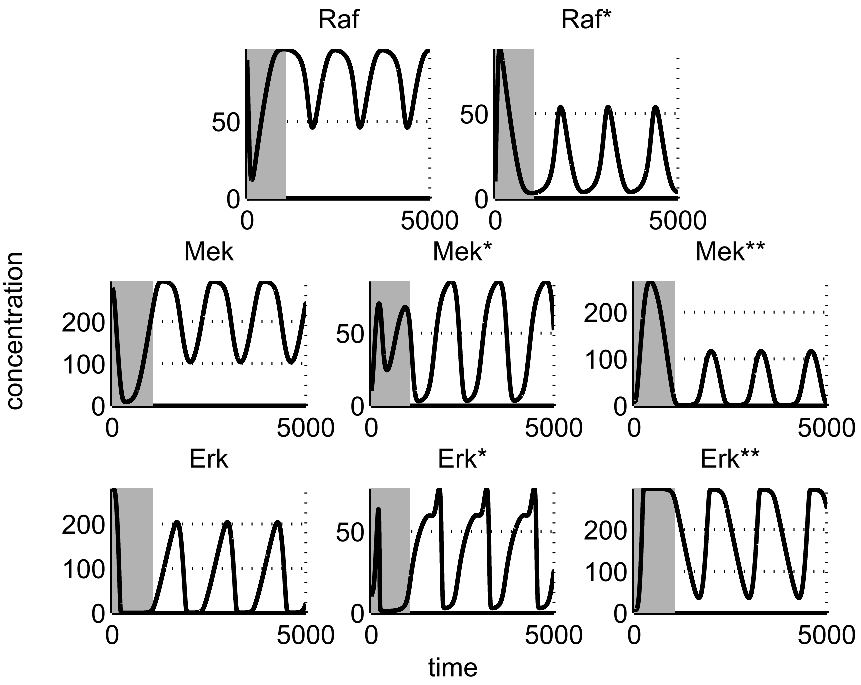

Figure 8: The long-term dynamics of the MAP kinase model shows regular oscillations. In our analysis, the first 1000 seconds, as highlighted by gray background color, have been analyzed as a compromise between a transient and oscillatory dynamics.

which is appropriate for validating the whole model is not informative for improving the model parameters. For such a purpose, new experimental data has to be generated for designs in which the model behavior is not yet precisely specified, i.e. for a setting with large prediction or validation confidence intervals. In these terms, Erk** (gray lines) is most informative in absolute units between 100 and 200 seconds. Also absolute measurements of Mek* (red lines) and Mek** (green lines) along the whole time axis are informative.

If only the amount of a phosphorylated form relative to the total concentration of the protein is experimentally accessible, then the panels on the right should be evaluated to assess the power of a design. In our example, the outcome for the prediction profile likelihood is very similar to the results obtained for absolute concentrations. Again, Erk for $t < 200$ as well as Mek* and Mek** are most informative. For the validation confidence intervals, Raf and Raf* appear as informative because the total concentration of Raf is three times smaller than the total amount of Mek and Erk.

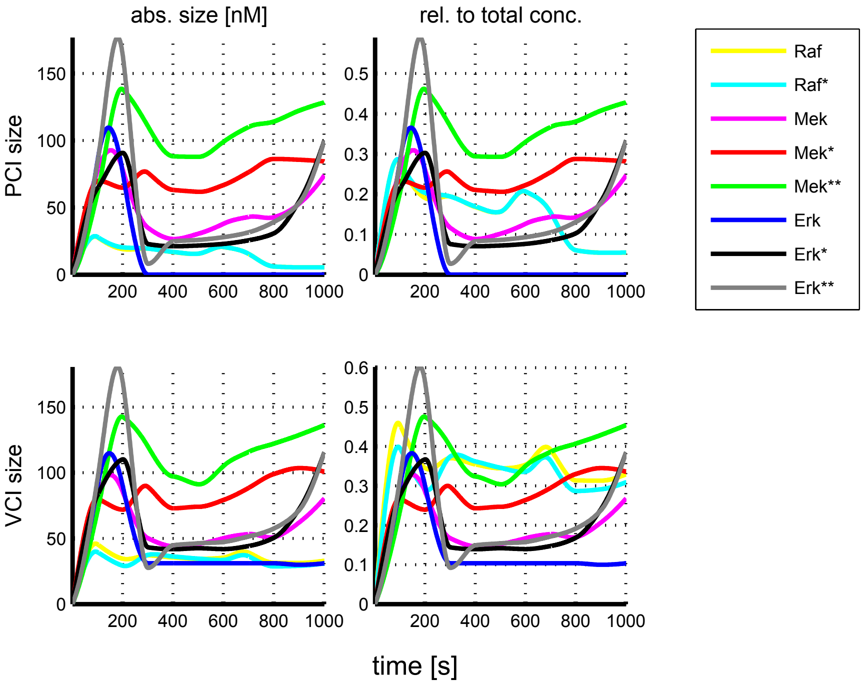

Figure 9: Size of the prediction confidence intervals for the dynamic states of the MAP kinase model. The left panels show the size of the confidence intervals in absolute units. In the panels on the right, the size is plotted relative to the total concentration of a protein. The upper row shows the results for the prediction confidence intervals, the lower row for the validation confidence intervals.

11 Implementation and Availability

The major task which has to be performed to apply the prediction profile likelihood approach is the application-specific implementation of the mathematical model as well as performing optimization to estimate parameters. Because this is highly context dependent there is difficult to provide the method in an universally applicable manner. Nevertheless, we provide four examples which can be used as templates to implement the prediction/validation profile likelihood calculation at http://www.fdmold.uni-freiburg.de/ckreutz/PPL.

Usually, a programming environment like R or MATLAB is utilized to simulate mathematical models. Such environments already provide optimization algorithms which can be used for parameter estimation. For calculating the prediction profile likelihood, the optimization has to be performed in a constraint manner as described in the main text. The computational implementation of the validation profile likelihood is even easier. In this case only validation data points have to be introduced and parameter estimation is performed comprehensively for the experimental and validation data. Equation (16) in the main text allows the calculation of the prediction profile likelihood from the validation profile, i.e. enables the calculation of

the PPL without constraint optimization. In the following, details about the implementation of our examples are provided.

We used the cvodes package from the Suite of Nonlinear Differential/Algebraic Equation Solvers (SUNDIALS) [5] for the numerical integration of the ODEs and the sensitivity equations. MATLAB's fmincon optimizer was used to estimate the parameters. The gradient and the Hessian of the objective function have been provided for the optimizer using the sensitivity equations [6] and the approximation

$$\frac{\partial^2}{\partial\theta_j\partial\theta_k}\mathrm{LL} \approx \sum_i \frac{1}{\sigma_i^2}\frac{\partial F_i}{\partial\theta_j}\frac{\partial F_i}{\partial\theta_k} \tag{34}$$

of the second derivatives [7]. Within a single optimization procedure, the parameters have been alternatingly optimized on the logarithmic scale as well as the common linear scale until the optimizer converged to a common value on both scales.

For the calculation of the validation profile likelihood, 30 test predictions z within the reasonable range of predictions given by the model structure and SD have been evaluated to obtain an initial guess of the likelihood shape. Then the grid is iteratively refined and/or enlarged until a smooth validation likelihood covering the whole confidence interval is obtained and local minima have been removed. For a single profile likelihood, around 10^2 optimizations were required. The numerical efficiency has been improved by using reasonable initial guesses based on previous estimates.

For the prediction profile likelihood the initial guess is obtained from the VPL by equation (16) in the main text. The gaps in this guess are then filled by nonlinear constrained optimization. If the constraint optimization procedure did not converge, the validation data error SD has been iteratively decreased by factors $10^0, 10^{-1}, \ldots, 10^{-5}$.

References

1. Raue A, Kreutz C, Maiwald T, Bachmann J, Schilling M, Klingmüller U, Timmer J: **Structural and practical identifiability analysis of partially observed dynamical models by exploiting the profile likelihood**. *Bioinformatics* 2009, **25**:1923–1929, [doi:10.1093/bioinformatics/btp358].

2. Cox D, Hinkley D: *Theoretical Statistics.* London: Chapman & Hall 1994.

3. Steiert B: **Identifiability Analysis and Experimental Design of Non-linear Dynamical Systems**. *Master's thesis*, Freiburg University, Freiburg, Germany 2012.

4. Kholodenko BN: **Negative feedback and ultrasensitivity can bring about oscillations in the mitogen-activated protein kinase cascades.** *Eur. J. Biochem.* 2000, **267**(6):1583–1588.

5. Hindmarsh AC, Brown PN, Grant KE, Lee SL, Serban R, Shumaker DE, Woodward CS: **SUNDIALS: Suite of nonlinear and differential/algebraic equation solvers**. *ACM T. Math. Softw.* 2005, **31**:363–396, [http://doi.acm.org/10.1145/1089014.1089020].

6. Leis J, Kramer M: **The simultaneous solution and sensitivity analysis of systems described by ordinary differential equations**. *ACM T. Math. Software* 1988, **14**:45–60.

7. Press W, Flannery B, Saul S, Vetterling W: *Numerical recipes.* Cambridge: Cambridge University Press 1992.
